# Do modest changes in skin temperature affect the measurement of micro- and macrovascular function?

**DOI:** 10.3389/fphys.2026.1812189

**Published:** 2026-06-17

**Authors:** Eva-Lotte Schabbehard, Franziska Riedl, Jasmin Anna Kienzner, Justin S. Lawley

**Affiliations:** Department of Sport Science, Division of Performance Physiology and Prevention, University of Innsbruck, Innsbruck, Austria

**Keywords:** cutaneous blood flow, flow-mediated dilation, forearm hemodynamics, local cooling, local heating, local hypoperfusion, reactive hyperaemia, retrograde shear rate

## Abstract

Cardiovascular disease is the leading cause of death worldwide, driven by risk factors such as atherosclerosis and endothelial dysfunction. Flow-mediated dilation (FMD), assessed as the percentage change in conduit artery vasodilation following local hypoperfusion, is a key tool to evaluate endothelial function. Microvascular changes, such as those induced by thermal stress, may influence baseline vascular conditions and potentially affect macrovascular responses during reactive hyperaemia. This study assesses the impact of skin blood flow on reactive hyperaemia and FMD. An initial FMD measurement was performed at each participant’s natural skin temperature (iFMD). After a 20-minute rest, skin temperature was randomly clamped to either 27 °C (cFMD) or 35 °C (wFMD). Baseline measurements revealed significantly higher skin temperature (*p* < 0.0001) and skin blood flow (*p* = 0.027) in wFMD compared to iFMD, and significantly lower skin temperature in cFMD (*p* < 0.0001). Retrograde shear rate was reduced in wFMD (*p* = 0.04). Cold skin temperature significantly reduced peak blood flow (*p* = 0.028), antegrade flow (*p* = 0.028), and conductance (*p* = 0.031). Peak values measured during reactive hyperaemia were similar between iFMD and wFMD, although skin blood flow was significantly higher in wFMD (*p* = 0.004). Despite these physiological changes, all calculated FMD values remained consistent across the three skin temperature conditions. Therefore, while FMD calculations appear robust to modest variations in skin temperature, differences in absolute values – particularly those related to skin blood flow – can influence the interpretation of FMD measurements and should be considered when comparing results across varying thermal conditions.

## Introduction

A sedentary lifestyle, smoking, obesity, diabetes mellitus, high total cholesterol, and hypertension (Torres et al., 2015; Shaito et al., 2022) are risk factors for atherosclerosis and endothelial dysfunction. This, in turn, can contribute to cardiovascular disease (CVD) (Incalza et al., 2018; Xu et al., 2021; Jebari-Benslaiman et al., 2022; Shaito et al., 2022; Baaten et al., 2023) which is the leading cause of death worldwide (Tsao et al., 2022). Endothelial dysfunction is characterized by a pro-inflammatory and pro-thrombotic phenotype of endothelial cells (Incalza et al., 2018; Xu et al., 2021; Jebari-Benslaiman et al., 2022). The result is an impaired flow-mediated dilation (FMD) response. A logical implication for reducing CVD is the early detection of endothelial dysfunction.

One non-invasive procedure that could potentially serve as a biomarker of CVD is the FMD assessment (Thijssen et al., 2019). This technique involves inflating a blood pressure cuff around a limb to cause a brief period (typically 3 to 5 minutes) of ischemia. Tissue hypoxia triggers the release of nitric oxide, prostaglandins, potassium, and adenosine (Rosenberry and Nelson, 2020), resulting in microvascular dilation and a decrease in vascular resistance distal to the occlusion cuff. Upon rapid cuff deflation, blood flow to the limb increases rapidly (reactive hyperaemia) in proportion to the downstream reduction in vascular resistance. Moreover, the increase in blood flow through the major conduit artery (typically the brachial or femoral artery) causes a rapid increase in shear stress and thus a “flow-mediated dilatation” (mechanotransduction) (Tremblay and Pyke, 2018; Sidnawi et al., 2021). Therefore, a reduced reactive hyperaemia indicates impaired microvascular dilator capacity, and a decrease in FMD reflects compromised macrovascular endothelial function, which serves as a predictor of CVD (Wu et al., 2015).

Consistent measurements of reactive hyperaemia and FMD are essential for their use as biomarkers of CVD (Hijmering et al., 2001). From the description provided, it is evident that the magnitude of FMD is dependent on two factors: one, the extent of reactive hyperaemia (i.e., the shear stress stimulus), which results from the degree of downstream vascular resistance induced by ischemia, and two, the local vasodilatory function of the conduit artery, mediated by both nitric oxide-dependent and -independent pathways. Moreover, several studies have shown that the magnitude of conduit artery dilation depends on the baseline diameter of the artery (Atkinson, 2014; Evanoff et al., 2016; Lolli et al., 2018; McLay et al., 2018). Studies (Pyke et al., 2004; Pyke and Tschakovsky, 2005) have suggested that normalizing FMD by shear rate, or the area under the curve (AUC) of shear rate, can eliminate the dependency on baseline diameter. Atkinson (2014) highlights the necessity of scaling FMD to baseline diameter to accurately interpret the dependence on diameter and shear rate.

Several strategies have been adopted to improve the measurement of reactive hyperaemia, such as estimating the degree of hypoxia caused by the ischemic period (Rosenberry et al., 2019) and correcting arterial dilation for the magnitude of shear stress (Pyke et al., 2004; Pyke and Tschakovsky, 2005; Tremblay and Pyke, 2018). However, one potential confounding factor that may impact the degree of reactive hyperaemia is a change in cutaneous vascular conductance. High ambient temperatures induce cutaneous vasodilation, a decrease in limb vascular resistance (i.e., lower basal vascular tone), and a subsequent increase in blood flow through the conduit artery. Conversely, cool ambient temperatures cause cutaneous vasoconstriction, an increase in limb vascular resistance (i.e., higher basal vascular tone), and a subsequent decrease in conduit artery flow. These physiological events may impact the FMD technique in two ways. First, changes in the basal tone of the cutaneous circulation may affect post-occlusive reactive hyperaemia. For example, under cool conditions, cutaneous blood vessels are “pre-constricted” prior to cuff inflation (Wilson et al., 2007). This hypothetically creates a large vascular bed for blood flow to enter when the occlusion cuff is released, as the FMD technique is not isolated to the skeletal muscle, in contrast to the dilated skin vasculature in hot conditions (Alali et al., 2020). Second, changes in resting blood flow through the conduit artery will cause a reciprocal change in resting conduit artery diameter, which is known to impact the calculation of FMD, as previously described.

Therefore, the primary aim of this study was to determine whether modest changes in cutaneous temperature and vascular resistance affect components of the FMD technique and whether current standardization procedures can account for these confounding factors. These temperatures were chosen to represent mild cooling and warming interventions, reflecting conditions such as entering the laboratory from a cooler or warmer environment or following light physical activity. To achieve this aim, the FMD technique was performed on three randomized occasions: under standardized laboratory conditions where forearm skin temperature and skin blood flow were free to vary on an individual basis (iFMD), and when forearm skin temperature was clamped to cool 27 °C (cFMD) and warm 35 °C (wFMD) conditions. We hypothesized that a major confounding factor would be changes in baseline diameter due to individual differences in skin blood flow under thermoneutral or warm conditions. Moreover, we expected that the baseline state of the cutaneous circulation would affect the absolute change in cutaneous vascular conductance during cuff release and influence the magnitude of vascular hyperaemia.

## Methods

### Participants

10 healthy participants (male = 6, female = 4) volunteered in this study. All participants were regularly physically active (8 ± 3 hours of exercise per week), non-smokers, and free of cardiac, pulmonary, or metabolic diseases. Male participants (age, 27 ± 4 years; height, 185 ± 9 cm; weight, 74.7 ± 6.5 kg; BMI, 21.8 ± 1 kg/m^2^) and female participants (age, 26 ± 4 years; height, 168 ± 7 cm; weight, 64.7 ± 8.7 kg; BMI, 22.8 ± 2.4 kg/m^2^) abstained from intense exercise, caffeine, and alcohol for 24 hours prior to all trials. Female participants were measured during the early follicular phase of their menstrual cycle. Each participant completed the visit with randomized assignment to the skin temperature interventions (see experimental protocol for details). Informed written consent was obtained after each participant received both verbal and written explanations of the experimental protocol and confirmed understanding of the potential risks involved. The study protocol was approved by the Ethics Committee of the University of Innsbruck and adhered to the principles of the Declaration of Helsinki.

### Experimental protocol

**Figure 1:**
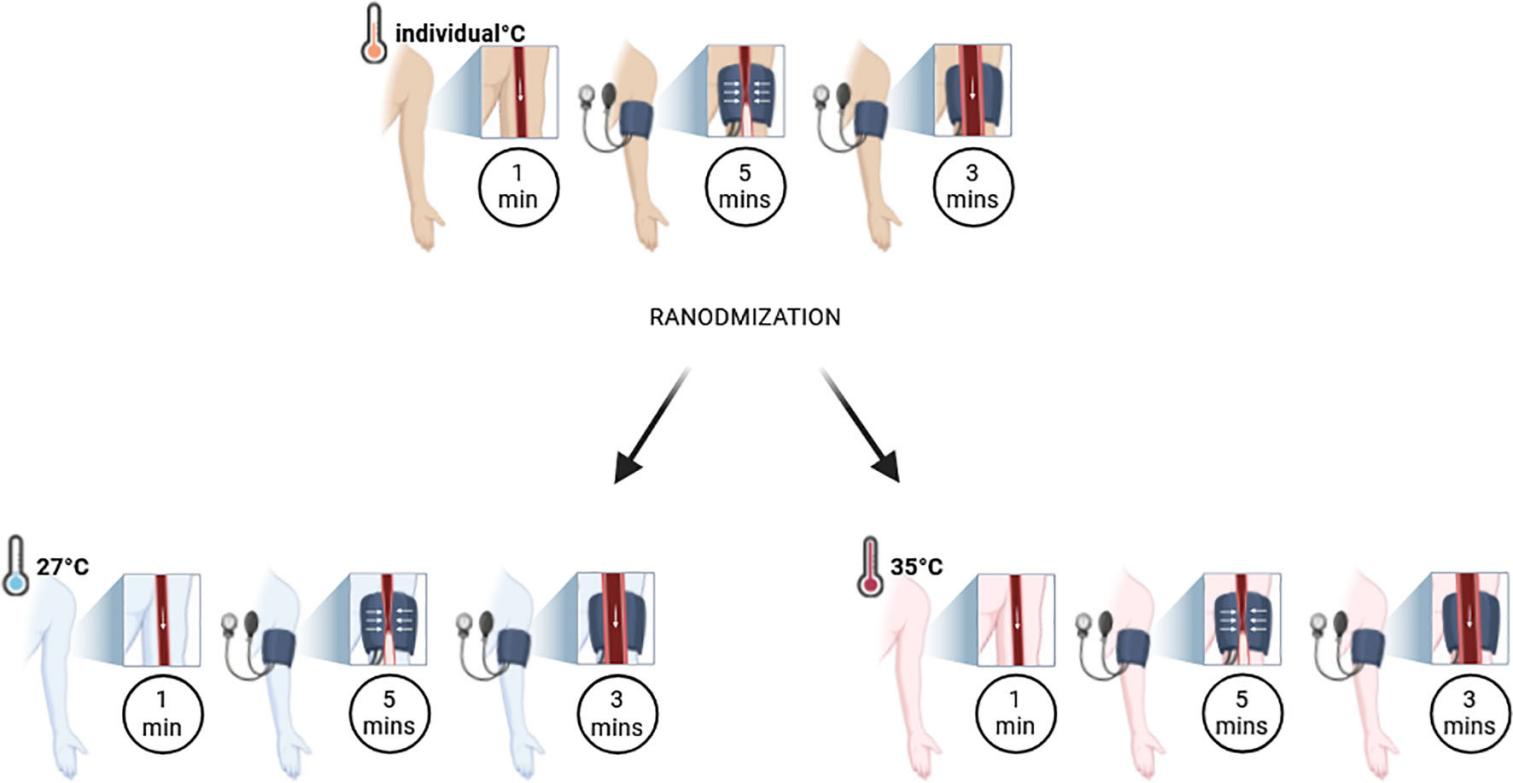
After an initial 20-minute stabilization in the supine position, baseline haemodynamic including brachial artery velocity and diameter were recorded over 1 minute. Thereafter, an occlusion cuff was inflated to 250 mmHg for 5 minutes and released. Haemodynamic including brachial artery diameter and velocity were monitored over 3 minutes of recovery. After a further 20 minutes recovery, the same procedure was performed in a randomized order when the forearm skin was cooled to 27°C or heated to 35°C. After another 20 minutes recovery period participants were exposed to the other temperature.

All measurements were conducted in the morning in a quiet, environmentally controlled physiology laboratory at the University of Innsbruck. Laboratory conditions were as follows: ambient temperature, 22.9 ± 1.1 °C; humidity, 46 ± 4%; barometric pressure, 948.9 ± 4.7 mmHg. After receiving ethical approval, participants’ body weight (Kern DS 150k1; Kern & Sohn, Germany) and height were measured. Participants were then seated in a semi-recumbent position (45°) on a hospital bed and equipped with the necessary sensors. FMD measurements were always conducted on the dominant arm. In this study, all participants were right-handed, and the left arm served as the control. Local skin blood flow was assessed using laser Doppler flowmetry. A purpose-built probe holder (PH2) was affixed to the ventral aspect of the forearm (mid-region) with great care to avoid contact with disruptive factors as skin hair or veins. A large-area optic probe (LP7A/T; 2 mm ring of collecting fibers) emitting laser light (wavelength 785 nm; intensity ~1 mW) was used to measure cutaneous red blood cell flux, expressed in perfusion units. The probe was inserted into the holder until gentle contact with the skin was achieved, with care taken to avoid cutaneous compression. The probe cable was secured to the skin using a strain relief loop to ensure consistent positioning and minimize movement artefacts throughout the experimental protocol. After a 20-minute rest period, an FMD measurement was performed under ambient conditions, i.e., at individual skin temperature (iFMD). Subsequently, in randomized order, additional FMD tests were conducted after skin temperature was clamped to either cool (27 °C; cFMD) or warm conditions (35 °C; wFMD). Before each FMD measurement, blood pressure was measured, with a 10-minute interval between assessments to minimize any potential effects on baseline FMD measurements. Skin temperature was manipulated using a water-perfused, tube-lined sleeve regulated by a circulating bath with a digital temperature controller (PolyScience, USA), and electrical heating sleeves controlled by a laboratory power supply unit (PeakTech^®^ 6225, Germany). FMD test were conducted once skin temperature had stabilized at the target level (heating: 10 ± 1 minutes; cooling: 8 ± 1 minutes). FMD tests were separated by a minimum of 20–30 minutes (Corretti et al., 2002), or until hemodynamic parameters were stable. Blood pressure was measured three times before each baseline FMD measurement. The FMD protocol followed the guidelines of Thijssen et al. (2019), consisting of a 1-minute baseline, 5-minute cuff occlusion at 250 mmHg, cuff release, and a 3-minute post-occlusion measurement (**Figure 1**).

### Experimental measurements

#### Thermal regulatory variables

Both forearm skin temperature and skin blood flow were continuously recorded using an integrated thermistor and a laser-Doppler flowmeter placed on the ventral forearm (Moor Instruments, Devon, UK). For the estimation of local skin blood flow, the large-area optic probe (LP7A/T, 2 mm ring of collecting fibers) emitted laser light (wavelength, 785 nm; intensity, ∼1 mW) onto the skin to measure cutaneous red cell flux and is presented as perfusion units. At baseline a purpose-built laser probe holder (PH2) is affixed to the skin. The probe is subsequently inserted into the probe holder until contact is made with the skin. Care is taken to avoid cutaneous compression. The probe wire is then affixed to the skin with a strain relief loop, such that the location and positioning of the probe are maintained throughout the entire experiment.

#### Cardiovascular variables

Heart rate was continuously recorded using three-lead electrocardiogram (Tram-rac, Solar 8000M GE, Marquette, USA). Blood pressure was measured intermittently on the left arm using an electrosphygmomanometry (Tango, M2, SunTechMedical Instruments Inc., USA) with a microphone placed over the brachial artery to detect Korotkoff sounds.

#### Ultrasonography

Brachial artery TAMV and diameter were measured using a 15L4A linear-array Doppler probe (15-MHz) (uSmart 3300, Terason, United States). simultaneously and in real time via custom-made wall-tracking and Doppler processing software. To ensure consistency, TAMV and diameter were measured at the same arterial segment for each test, using anatomical landmarks during B-mode imaging. TAMV was recorded at an insonation angle of 60° and simultaneously imported, along with diameter values, into LabChart via the Doppler processing software. Antegrade and retrograde TAMV data were captured in separate LabChart channels. All measurements were performed by the same investigator, and ultrasound settings including depth, gain, power, dynamic range, and sample volume were kept constant for each participant throughout the study.

### Data acquisition and analysis

All continuous measurements were sampled at 250 Hz using a Powerlab system (Powerlab; AD Instruments, Oxford, UK) and extracted via an offline data acquisition software (LabChart 8; AD Instruments, Oxford, UK). Skin temperature and heart rate were extracted as 60 second averages during the baseline FMD and as 120-seconds averages following cuff release. Ultrasound and skin blood flow data were continuously recorded and extracted as: 1. the mean of 60-seconds during baseline, 2. the average of the three highest consecutive peak values following cuff release (i.e., peak blood flow), 3. the AUC over the first 30 seconds post cuff release (AUC-30), and 4. the AUC from cuff release to the peak change in arterial diameter (AUC-peak diameter). Mean arterial pressure (mmHg) (Equation 1), blood flow (ml·min^-1^) (Equation 2), shear rate (s^-1^) (Equation 3), and conductance (ml·min^-1^·mmHg^-1^) (Equation 4) were calculated as follows. The absolute percent change in diameter during reactive hyperaemia (FMD %) is presented in Equation 5. Equation 6 presents the FMD with peak shear rate during reactive hyperaemia. The AUC for shear rate, from cuff release to the time point when peak diameter is reached (shear rate _AUC-peak diameter_) was calculated, using the trapezoidal rule (Padilla et al., 2008; Iwamoto et al., 2018; Alali et al., 2020) (Equation 7). Additionally, the AUC from cuff release to 30 seconds post-release (AUC_30-seconds_) (Equation 9) is calculated. In this context, x represents time, y represents shear rate, and z represents baseline shear rate. Both results were used to normalized FMD (Equations 8, 10).

(1) mean arterial pressure=2/3 diastolic blood pressure+1/3 systolic blood pressure

(2) $$\text{blood flow}=\text{TAMV}\cdot \text{π}{\left(\frac{\text{diameter}}{2}\right)}^{2}\cdot 60$$

(3) $$\text{shear rate}=4\text{x}(\frac{\text{TAMV}}{\text{diameter}})$$

(4) $$\text{conductance}=\frac{\text{blood flow}}{\text{mean arterial pressure}}$$

(5) $$\text{FMD }(\%)=\frac{\text{peak diameter - baseline diameter}}{\text{baseline diameter}}\cdot 100$$

(6) $${\text{FMD}}_{\text{peak shear rate}}=\frac{\text{FMD }(\%)}{\text{peak shear rate}}$$

(7) $${\text{Shear rate}}_{\text{AUC}-\text{peak diameter}}=\sum_{\text{i}=1}^{\text{peak diameter}}[\frac{1}{2}\cdot ({\text{x}}_{\left(\text{i}+1\right)}-{\text{x}}_{\text{i}})\cdot ({\text{y}}_{\left(\text{i}+1\right)}-{\text{y}}_{\text{i}})+({\text{x}}_{\left(\text{i}+1\right)}-{\text{x}}_{\text{i}})\cdot ({\text{y}}_{\text{i}}-\text{z})]$$

(8) $${\text{FMD}}_{\text{AUC}-\text{peak diameter}}=\frac{\text{FMD}\;(\%)}{{\text{shear rate}}_{\text{AUC}-\text{peak diameter}}}$$

(9) $${\text{Shear rate}}_{\text{AUC}-30\text{ seconds}}=\sum_{\text{i}=1}^{\text{diameter }30-\text{seconds post}-\text{release}}[\frac{1}{2}\cdot ({\text{x}}_{\left(\text{i}+1\right)}-{\text{x}}_{\text{i}})\cdot ({\text{y}}_{\left(\text{i}+1\right)}-{\text{y}}_{\text{i}})+({\text{x}}_{\left(\text{i}+1\right)}-{\text{x}}_{\text{i}})\cdot ({\text{y}}_{\text{i}}-\text{z})]$$

(10) $${\text{FMD}}_{\text{AUC}-30\text{ seconds}}=\frac{\text{FMD}\;(\%)}{{\text{shear rate}}_{\text{AUC}-30\text{ seconds}}}$$

### Power calculation

To determine sample size, we estimated theoretically important physiological differences in reactive hyperemia peak blood flow (Δ 50 ml·min^-1^) and brachial diameter (Δ 0.2cm) post cuff release. For peak blood flow a change of 50 ml·min^-1^ with a pooled standardization of 100 ml·min^-1^ resulted in an effect size of 1.11 and estimated sample size of between 9 (80% power) and 13 (95% power) participants. For a change in peak diameter, a 0.2 cm change with a pooled standard deviation of 0.04 cm (Torres et al., 2015) resulted in an effect size of 1.12 and an estimated sample size of between 9 (80% power) and 13 (95% power) participants. All power calculation was based on an alpha probability of 0.05 using G* Power 3.1.9.7.

### Statistical analyses

To assess the influence of skin temperature on micro- and macrovascular function, a repeated-measurement ANOVA (iFMD vs. cFMD vs. wFMD) was performed for each variable. Subsequently, a Holm-Šídák multiple comparisons test was applied to compare groups after a significant main effect. Values are expressed as mean ± standard deviation, with statistical significance set at *p* ≤ 0.05. Additionally, the percentage change between baseline and reactive hyperemia was calculated for each FMD measurement using the following formula: ((peak – baseline)/baseline) · 100. The results are resented in **Table 1**. All analyses were conducted using Prism 9 (GraphPad Software Inc., La Jolla, CA) including the Shapiro-Wilk test for normality.

**Table 1:** Percentage changes from FMD-baseline to reactive hyperemia for all variables measured during each FMD assessment (individual skin temperature, cooled, and heated conditions).

	Individual	Cold exposed	Heat exposed
Skin temperature (°C)	-0.07 ± 0.56%	-2.52 ± 1.45%	0.03 ± 3.08%
Control skin temperature (°C)	-0.08 ± 0.62%	-0.39 ± 0.81%	-0.36 ± 0.43%
Skin blood flow (PU)	213.01 ± 118.39%	174.21 ± 131.06%	78.84 ± 87.95%
Control skin blood flow (PU)	-3.83 ± 8.18%	4.6 ± 22.64%	4.71 ± 14.86%
Heart rate (bpm)	-2.02 ± 3.55%	-0.73 ± 7.47	0.89 ± 3.34%
TAMV (cm·s^-1^)	1288.98 ± 391.26%	1420.89 ± 612.18%	1132.24 ± 484.14%
Antegrade TAMV (cm·s^-1^)	1126.95 ± 385.8%	1138.2 ± 432.35%	1044.365 ± 435.5%
Retrograde TAMV (cm·s^-1^)	-96.18 ± 7.37%	-99.48 ± 0.7%	-96.332 ± 5.94%
Diameter (cm)	6.32 ± 2.72%	5.46 ± 2.3%	7.33 ± 3.79%
Blood flow (ml·min^-1^)	1279.99 ± 385.04%	1379.79 ± 570.83%	1151.03 ± 476.97%
Antegrade blood flow (ml·min^-1^)	1121.95 ± 393.97%	1110.61 ± 429.12%	1062.42 ± 430.99%
Retrograde blood flow (ml·min^-1^)	-96.17 ± 7.41%	-99.504 ± 0.66%	-96.42 ± 5.55%
Shear rate (s^-1^)	1294.06 ± 397.16%	1442.97 ± 636.81%	1127.17 ± 500.72%
Antegrade shear rate (s^-1^)	1129.97 ± 383.92%	1153.05 ± 435.82%	1039.43 ± 450.69%
Retrograde shear rate (s^-1^)	-96.18 ± 7.35%	-99.46 ± 0.72%	-96.27 ± 6.2%
Conductance (ml·min^-1^·mmHg^-1^)	-92.14 ± 2.27%	-91.89 ± 3.8	-89.74 ± 7.15%

All values are expressed as mean ± standard deviation.

## Results

### The effect of skin temperature on microvascular and macrovascular function

In comparison to the thermoneutral condition, baseline skin temperature was significantly lower in the cFMD (*p* < 0.0001) condition and significantly higher in the wFMD (*p* < 0.0001) condition. Moreover, skin temperature was significantly lower in the cFMD compared to the wFMD condition (*p* < 0.0001). Skin blood flow was significantly higher in the wFMD condition compared to both iFMD (*p* = 0.027) and cFMD (*p* = 0.048). Skin blood flow showed no difference between iFMD and cFMD (*p* = 0.736). Moreover, there was a small effect on retrograde shear rate between iFMD and wFMD (*p* = 0.040) and cFMD and wFMD (*p* = 0.031). There were no other main effects for baseline diameter, antegrade and retrograde TAMV, blood flow, antegrade shear rate, and conductance.

**Figure 2:**
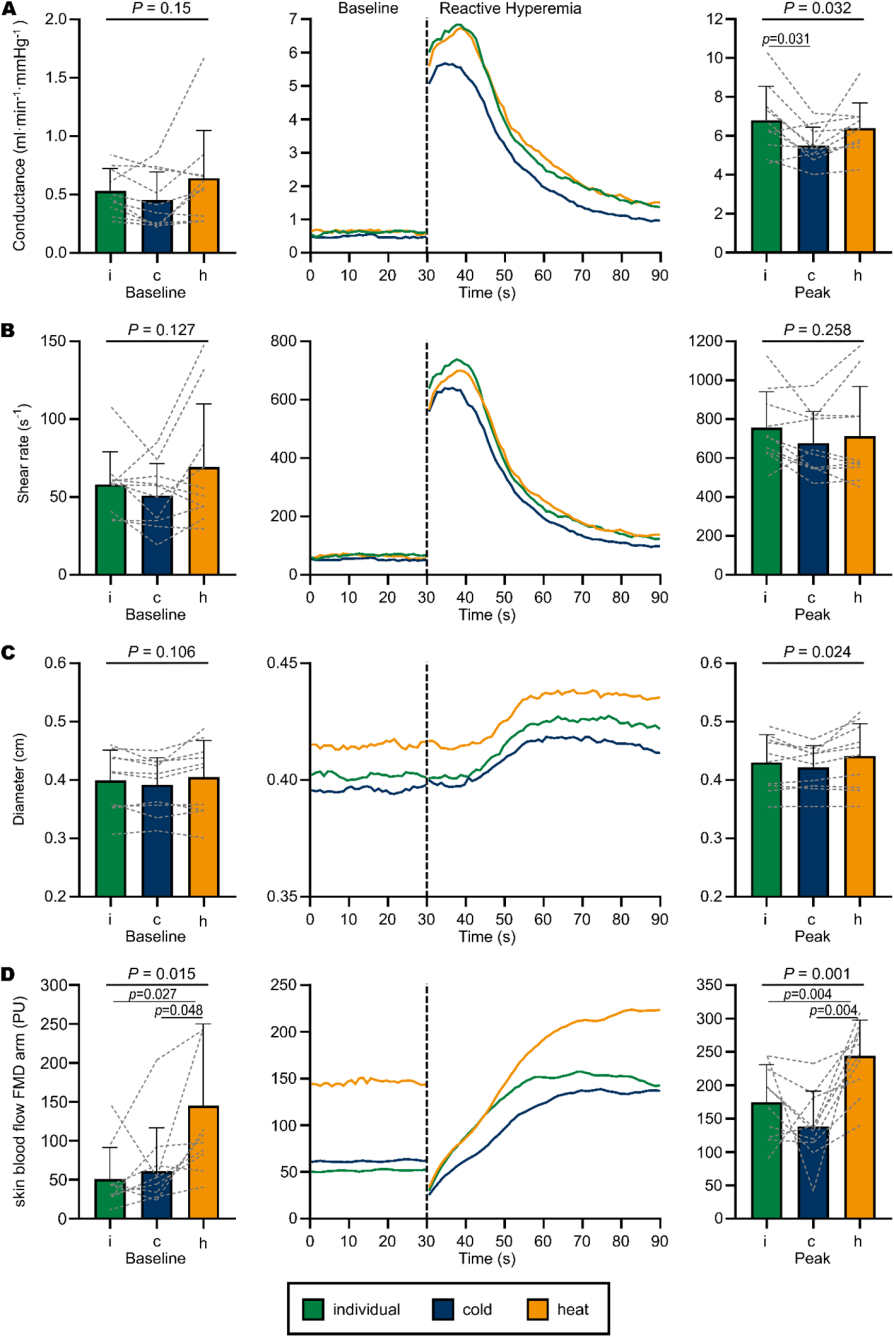
Comparison of baseline and peak mean values for three FMD measurements conducted at individual (green), cooled (27°C; blue), and heated (35°C; orange) skin temperatures. The illustrated variables include **(A)** conductance, **(B)** shear rate, **(C)** diameter, and **(D)** skin blood flow. Continuous data (mean per second) is presented for baseline (0 to 30 seconds) and reactive hyperaemia (30 to 90 seconds). *P*-values (capital *P*) above the graphs indicate the results of the repeated-measurement ANOVA, while *p*-values (lowercase *p*) above the bar represented Holm-Šídák multiple comparisons. For visual representation on individual data, reactive hyperaemia values were either accumulated (for heart rates above 60 bpm) or extended (for heart rates below 60 bpm) to produce 60 values for each post occlusive reactive hyperaemia event.

**Figure 3:**
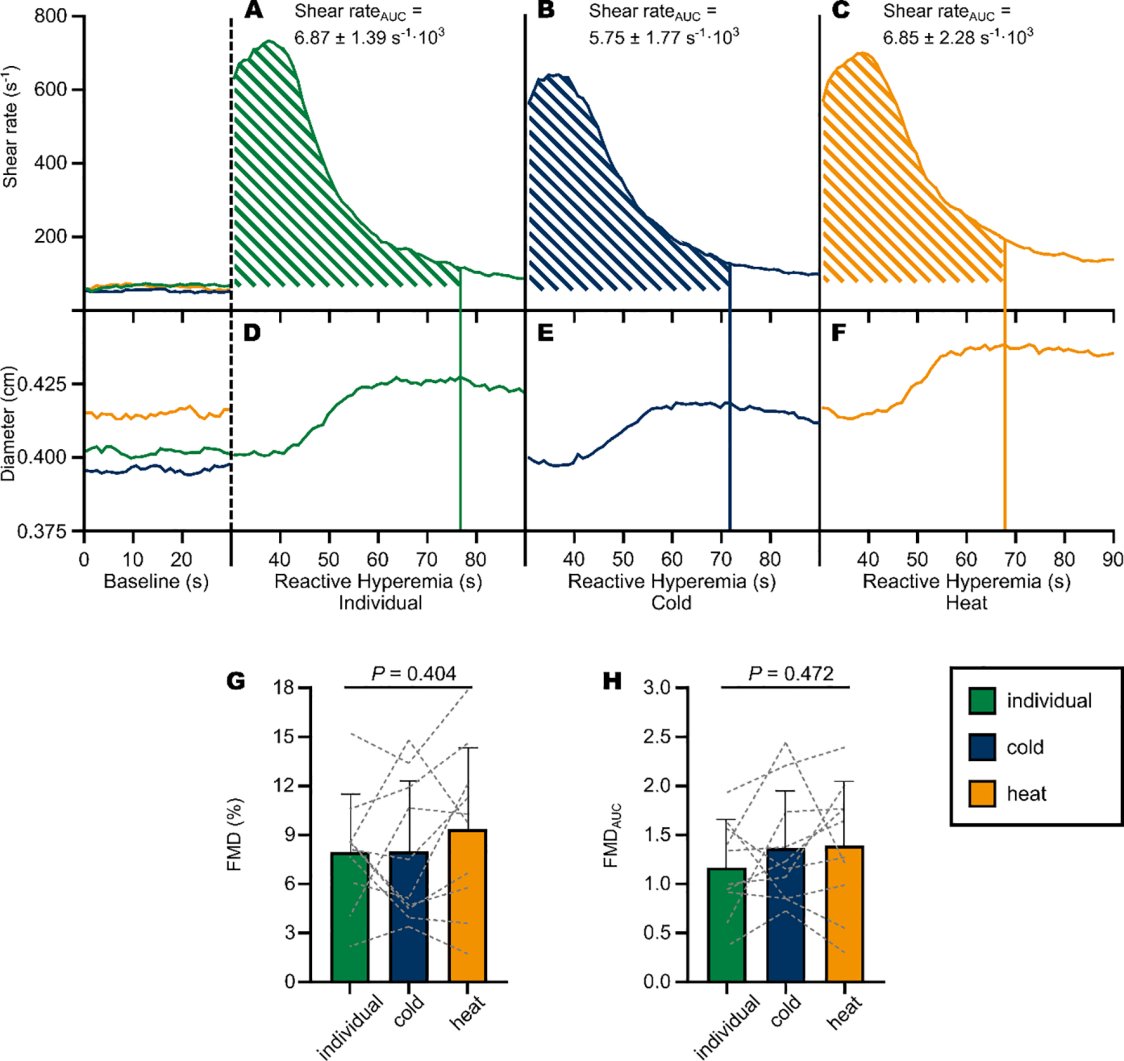
Continuous data (mean per second) is presented in the first 30 seconds for both shear rate and diameter across the three FMD measurements: individual (green), cooled (27°C; blue), and heated (35°C; orange) skin temperatures. The area under the curve (AUC) for shear rate is calculated from cuff release to time when the peak diameter is reached to baseline shear and shear rate value for each second. AUC is shown for **(A)** the individual FMD, **(B)** the cooled skin temperature FMD, and **(C)** the heated skin temperature FMD. Continuous diameter values during reactive hyperaemia are demonstrated for **(D)** the individual FMD, **(E)** the cooled skin temperature FMD, and **(F)** the heated skin temperature FMD with lines indicating peak diameters. The **(G)** mean FMD (%) is calculated between the absolute peak diameter (mean for three consecutive heart beats) and the absolute baseline diameter (mean for three consecutive heart beats). **(H)** FMD was normalized with the AUC of shear rate. *P*-values reflect the results of the repeated-measurement ANOVA.

After cuff release, skin blood flow remained higher in the wFMD condition compared to both the iFMD (*p* = 0.004) and cFMD (*p* = 0.004). No difference was found between iFMD and cFMD for skin blood flow (*p* = 0.171). There was a main effect for the peak diameter (ANOVA, *p* = 0.024), albeit differences between groups failed to reach the statistical threshold (**Table 2**). Moreover, peak blood flow (*p* = 0.028), antegrade blood flow (*p* = 0.028), and conductance (*p* = 0.031) were significantly lower in cFMD versus wFMD and iFMD conditions. No statistical differences were found for antegrade and retrograde TAMV and shear rate. FMD indices were similar across all three skin temperature conditions (**Figures 2**, **3**; **Tables 2**, **3**).

**Table 2:** Data for three FMD measurements with different skin temperatures: individual, cooled (27°C), and heated (35°C).

	Individual	Cold exposed	Heat exposed	*P* value
Skin temperature (°C)	30.57 ± 0.82	26.91 ± 0.69	35.98 ± 1.01	**<0.0001**
Control skin temperature (°C)	30.73 ± 0.88	30.67 ± 1.03	30.75 ± 1.07	0.747
Mean arterial pressure (mmHg)	81 ± 7	82 ± 5	83 ± 5	0.489
Heart rate (bpm)	57 ± 9	55 ± 9	54 ± 8	0.157
Baseline				
TAMV (cm·s^-1^)	5.67 ± 1.69	4.97 ± 2.13	6.82 ± 3.91	0.133
Antegrade TAMV (cm·s^-1^)	6.45 ± 1.8	5.89 ± 2.31	7.15 ± 3.75	0.343
Retrograde TAMV (cm·s^-1^)	-0.72 ± 0.78	-0.95 ± 0.83	-0.32 ± 0.26	**0.023**
Diameter (cm)	0.4 ± 0.05	0.39 ± 0.05	0.41 ± 0.06	0.106
Blood flow (ml·min^-1^)	42.55 ± 13.88	37.02 ± 20.02	52.67 ± 32.79	0.133
Antegrade blood flow (ml·min^-1^)	48.34 ± 18.66	44.33 ± 23.26	55.6 ± 32.26	0.28
Retrograde blood flow (ml·min^-1^)	-6.83 ± 7.01	-7.53 ± 7.7	-2.85 ± 2.63	**0.031**
Shear rate (s^-1^)	58.11 ± 20.82	50.95 ± 20.62	69.28 ± 40.58	0.127
Antegrade shear rate (s^-1^)	64.04 ± 20.11	60.09 ± 21.38	72.42 ± 38.91	0.323
Retrograde shear rate (s^-1^)	-7.78 ± 6.73	-9.43 ± 7.49	-3.06 ± 2.25	**0.007**
Conductance (ml·min^-1^·mmHg^-1^)	0.53 ± 0.19	0.45 ± 0.24	0.64 ± 0.45	0.15
Skin blood flow (PU)	50.96 ± 40.48	46.01 ± 23.91	145.2 ± 104.8	**0.015**
Control skin blood flow (PU)	30.5 ± 18.85	26.76 ± 15.07	25.71 ± 14.73	0.1
Peak				
TAMV (cm·s^-1^)	73.45 ± 9.68	64.17 ± 10.14	69.38 ± 15.2	0.074
Antegrade TAMV (cm·s^-1^)	73.45 ± 9.68	64.17 ± 10.14	69.38 ± 15.20	0.074
Retrograde TAMV (cm·s^-1^)	-0.01 ± 0.01	-0.01 ± 0.01	-0.002 ± 0.003	0.238
Diameter (cm)	0.43 ± .05	0.42 ± 0.04	0.44 ± 0.06	**0.024**
Blood flow (ml·min^-1^)	550 ± 133.9	450.2 ± 86.78	528.6 ± 101.8	**0.011**
Antegrade blood flow (ml·min^-1^)	550 ± 133.9	450.2 ± 86.78	528.6 ± 101.8	**0.011**
Retrograde blood flow (ml·min^-1^)	-0.05 ± 0.06	-0.06 ± 0.09	-0.01 ± 0.01	0.182
Shear rate (s^-1^)	756.6 ± 183.8	677.4 ± 162.1	712.3 ± 255	0.258
Antegrade shear rate (s^-1^)	756.6 ± 183.8	677.4 ± 162.1	712.3 ± 255	0.258
Retrograde shear rate (s^-1^)	-0.05 ± 0.06	-0.07 ± 0.11	-0.02 ± 0.03	0.289
Conductance (ml·min^-1^·mmHg^-1^)	6.79 ± 1.76	5.51 ± 0.93	6.39 ± 1.3	**0.032**
Skin blood flow (PU)	174.6 ± 56.37	138.4 ± 52.93	244 ± 53.67	**0.001**
Control skin blood flow (PU)	29.36 ± 19.7	29.49 ± 22.89	26.81 ± 17.07	0.321
FMD (%)	7.96 ± 3.55	8 ± 4.31	9.36 ± 4.98	0.404
FMD/peak shear rate	0.011 ± 0.004	0.011 ± 0.005	0.014 ± 0.007	0.334
Shear rate_AUC-peak diameter_ (s^-1^·10^3^)	6.87 ± 1.39	5.75 ± 1.77	6.85 ± 2.28	0.111
FMD_AUC-peak diameter_	1.17 ± 0.49	1.37 ± 0.59	1.39 ± 0.65	0.472
Shear rate_AUC-30 seconds_ (s^-1^·10^3^)	6.72 ± 1.45	5.79 ± 1.76	6.48 ± 2.19	0.13
FMD_AUC-30 seconds_	1.2 ± 0.49	1.34 ± 0.52	1.47 ± 0.71	0.466

Continiously measured variables include intervention and control arm skin temperature and skin blood flow, mean arterial pressure and heart rate. Baseline values are a mean of 60 seconds and peak values are a mean of three consecutive heart beats.

All values are expressed as mean ± standard deviation; AUC, area under the curve; TAMV, time average mean velocity; *P*-values reflect the results of the repeated-measurement ANOVA with a significance level of 0.05.

The values in bold are statistically significant (p < 0.05).

**Table 3:** Presented are Holm-Šídák multiple comparisons calculated within a repeated measurement ANOVA.

	Individual vs. cold	Individual vs. heated	Cold vs. heated
Skin temperature (°C)	**<0.0001**	**<0.0001**	**<0.0001**
Control skin temperature (°C)	0.906	0.932	0.687
Mean arterial pressure (mmHg)	0.914	0.564	0.551
Heart rate (bpm)	0.394	**0.034**	0.741
Baseline			
TAMV (cm·s^-1^)	0.383	0.383	0.177
Antegrade TAMV (cm·s^-1^)	0.518	0.519	0.502
Retrograde TAMV (cm·s^-1^)	0.291	0.149	0.051
Diameter (cm)	0.235	0.321	0.235
Blood flow (ml·min^-1^)	0.323	0.323	0.159
Antegrade blood flow (ml·min^-1^)	0.455	0.455	0.423
Retrograde blood flow (ml·min^-1^)	0.561	0.107	0.107
Shear rate (s^-1^)	0.414	0.414	0.2
Antegrade shear rate (s^-1^)	0.632	0.632	0.536
Retrograde shear rate (s^-1^)	0.238	**0.04**	0.314
Conductance (ml·min^-1^·mmHg^-1^)	0.199	0.348	0.177
Skin blood flow (PU)	0.736	**0.027**	**0.048**
Control skin blood flow (PU)	0.189	0.177	0.653
Peak			
TAMV (cm·s^-1^)	0.064	0.381	0.189
Antegrade TAMV (cm·s^-1^)	0.064	0.381	0.189
Retrograde TAMV (cm·s^-1^)	0.496	0.463	0.463
Diameter (cm)	0.166	0.166	0.075
Blood flow (ml·min^-1^)	**0.028**	0.519	**0.053**
Antegrade blood flow (ml·min^-1^)	**0.028**	0.519	**0.053**
Retrograde blood flow (ml·min^-1^)	0.489	0.4	0.4
Shear rate (s^-1^)	0.204	0.626	0.626
Antegrade shear rate (s^-1^)	0.204	0.626	0.626
Retrograde shear rate (s^-1^)	0.572	0.572	0.572
Conductance (ml·min^-1^·mmHg^-1^)	**0.031**	0.456	0.071
Skin blood flow (PU)	0.171	**0.004**	**0.004**
Control skin blood flow (PU)	0.944	0.438	0.478
FMD (%)	0.98	0.56	0.56
FMD/peak shear rate	0.679	0.529	0.529
Shear rate_AUC-peak diameter_ (s^-1^·10^3^)	0.082	0.98	0.053
FMD_AUC-peak diameter_	0.679	0.679	0.891
Shear rate_AUC-30 seconds_ (s^-1^·10^3^)	0.098	0.682	0.108
FMD_AUC-30 seconds_	0.759	0.629	0.759

Variables include three FMD measurements with different skin temperatures: individual, cooled (27°C), and heated (35°C). Continiously measured variables include intervention and control arm skin temperature and skin blood flow, mean arterial pressure and heart rate. Baseline values are a mean of 60 seconds and peak values are a mean of three consecutive heart beats.

AUC, area under the curve; TAMV, time average mean velocity; *p*-values reflect the results of Holm-Šídák multiple comparisons calculated within a repeated measurement ANOVA with a significance level of 0.05. The values in bold were significant values.

## Discussion

This study investigated the influence of modest alterations in skin temperature on the quantification of micro- and macrovascular function. Despite significant alterations in skin temperature and corresponding changes in skin blood flow (observed during heating only), retrograde shear rate, and conductance, these factors did not translate into differences in FMD outcomes when expressed relative to the shear stimulus.

Interestingly, relatively mild local forearm cooling caused an increase in resting retrograde shear rate and an attenuated hyperaemic response to cuff ischemia, despite no significant changes in cutaneous blood flow. These findings suggest that FMD metrics are robust to modest changes in skin temperature and skin blood flow, if baseline changes in arterial diameter and reactive hyperaemia are taken into account. Nonetheless, prolonged periods of active skin cooling should be avoided due to its attenuating effect on the hyperaemic response, which occurs independently of changes in skin blood flow and may compromise the accurate quantification of microvascular function.

### The effect of increasing skin temperature on microvascular and macrovascular function

The ischemic cuff technique is a well-established method to measure microvascular function through the increase in blood flow following a period of limb ischemia, and macrovascular function via the resulting increase in conduit artery dilation (i.e., flow-mediated dilation) (Thijssen et al., 2019; Rosenberry and Nelson, 2020). Changes in skin temperature are known to increase skin blood flow, which impacts forearm haemodynamics (Nagasaka et al., 1987). Indeed, it is common during hemodynamic and pharmacological studies to apply a fan directed towards the forearm to limit these effects (Crecelius et al., 2015; Hearon et al., 2020; Hansen et al., 2022).

In the current study, a modest increase in skin temperature and skin blood flow had a minor impact on resting and reactive hyperaemic hemodynamic metrics. For example, while baseline skin blood flow was elevated during forearm heating, this caused a small, non-statistically significant increase in resting shear rate (11.17 s^-1^) and arterial diameter (0.1 cm). Moreover, upon cuff release, the absolute increase from baseline to peak in forearm skin blood flow (baseline iFMD 50.96 PU vs. peak iFMD 174.6 PU; baseline wFMD 145.2 PU vs. peak wFMD 244 PU) and brachial shear rate (baseline iFMD 58.11 s^-1^ vs. peak iFMD 756.6 s^-1^; baseline wFMD 69.28 s^-1^ vs. peak wFMD 712.3 s^-1^) were of similar magnitude, resulting in a similar degree of flow-mediated dilation between conditions.

Previous studies have observed that elevated skin temperatures augment the cutaneous (Abraham et al., 2013), microvascular, and conduit artery vasodilatory response to ischemia (Cheng et al., 2019; Coombs et al., 2021). The difference between the current study and the three previously cited studies is likely due to the interaction of local vs. whole body heating and the magnitude of heat stress. For example, while only heating the forearm, the protocol by Coombs et al. (2021) involved ~60 minutes at a skin temperature of ~38°C, compared to only ~10 minutes at ~36°C in the current investigation. Similar improvements with isolated forearm heating have been observed with 10 minutes at a skin temperature of ~42°C.

We observed that local skin cooling blunted, and local warming augmented the cutaneous hyperaemic response following ischemia. Indeed, while the study by Abraham et al. (2013) manipulated skin temperature by altering ambient temperature, skin temperatures in the low and high room temperature conditions were comparable to the current study. Their data showed blunted and augmented cutaneous responses to acetylcholine, sodium nitroprusside, and ischemia in low and high room temperatures, respectively.

### The effect of decreasing skin temperature on microvascular and macrovascular function

In the current study, skin cooling was quite modest (Δ3.66°C), yet it caused a significant increase in resting retrograde blood flow. Moreover, upon cuff release, the hyperaemic response was attenuated, as indicated by a lower brachial blood flow and vascular conductance after cuff release.

Interestingly, two lines of evidence suggest that this effect is not due to changes in cutaneous blood flow. First, unlike passive heating, mild local cold stress did not significantly reduce cutaneous blood flow at rest. Secondly, examining the time course data (**Figure 2**) shows that blood flow through the brachial artery peaks at ~30 seconds, at which time skin blood flow is relatively unchanged, yet thereafter progressively increases over time, similar to the dilation of the brachial artery. It seems that changing the basal tone of the cutaneous circulation mainly affects flow-mediated dilation of the cutaneous circulation after the hyperaemic event, and not vice versa.

The reason why mild cutaneous cooling increased resting retrograde shear rate and suppressed the reactive hyperaemia is unknown. Yet, given the observation that resting skin blood flow was unchanged, the most likely explanation is a slight cooling of the skeletal muscle. With skeletal muscle cooling, resting muscle metabolism is reduced (Jones et al., 2024). As such, the hypoxic stimulus – and thus likely metabolic production and metabolite accumulation – is reduced during cuff inflation, which has been shown to be a major predictor of the hyperaemic response (Yu et al., 2005).

Resting retrograde blood flow and shear rate are important parameters when examining forearm hemodynamics and downstream vascular resistance (Thijssen et al., 2019). Moreover, post-ischemic reactive hyperaemia is a marker of forearm microvascular function (Coccarelli and Nelson, 2023). Thus, while typical techniques such as applying a fan directed at the forearm are likely a good experimental choice to control periods of elevated skin blood flow, active cooling of the skin – even by ~3 °C – should be avoided.

### Limitations

One limitation of this study is the use of laser-Doppler flowmetry to assess skin blood flow. The standard deviation of skin blood flow in the experimental and control arms was high, indicating heterogeneous cutaneous responses to skin temperature. Yet, the responses should be highly reliable, as once the laser probe was fixed to the ventral forearm, it remained in place throughout the study.

Another limitation is the relatively modest changes in skin temperature and the short cooling and warming periods. Nonetheless, this study aimed to examine subtle changes in skin temperature that accompany ambient fluctuations in laboratories, field studies, or experimental manipulations to control skin temperature in hemodynamic and pharmacological research.

Finally, an obvious limitation is the inclusion of relatively young individuals, who were mainly sports science students and thus relatively well-trained. Skin blood flow is dependent on fitness level and can be altered by endurance exercise (Cramer et al., 2022). Furthermore, skin blood flow and flow-mediated dilation are influenced by age and clinical diagnosis (Benjamin et al., 2004; Holowatz et al., 2010), which can affect skin blood flow levels between subjects with similar skin temperature (Simmons et al., 2011). While more research is conceivably needed to understand how modest changes in skin temperature affect the FMD technique in these populations, it is unlikely that such mild changes would affect the FMD response, because the cutaneous responses were delayed relative to the hyperaemic blood flow response.

### Practical implications

These data indicate that even modest changes in skin temperature and skin blood flow can influence the measurement of flow-mediated dilation. Techniques such as using a light fan or a damp towel on the forearm to limit the effects of elevated skin blood flow on resting brachial blood flow and shear rate are likely appropriate for studies measuring vascular function (e.g., FMD or handgrip exercise). However, active cooling or heating of the forearm is unlikely to improve the standardization of these measurements.

## Conclusion

The present findings demonstrate that modest changes in skin temperature and the associated alterations in skin blood flow can influence baseline hemodynamic conditions relevant to the assessment of flow-mediated dilation (FMD). However, when the shear stimulus is appropriately accounted for, the overall quantification of FMD remains largely preserved, indicating that the dilation response primarily reflects endothelial sensitivity to shear stress. These results suggest that strict clamping of skin temperature may not be necessary for the interpretation of FMD outcomes. Nevertheless, minimizing variability in skin blood flow—through simple approaches such as maintaining stable environmental conditions or using passive techniques (e.g., airflow or dampening of the skin)—may help improve measurement consistency. In contrast, active manipulation of forearm temperature, such as external heating or cooling, does not appear to enhance standardization and may introduce additional alterations in hemodynamic responses, including changes in retrograde flow and reactive hyperaemia. Therefore, such interventions should be applied with caution when assessing vascular function.

## Data Availability

The original contributions presented in the study are included in the article/supplementary material. Further inquiries can be directed to the corresponding author.
